# Neuroprotective Mechanism of *Lycium barbarum* Polysaccharides against Hippocampal-Dependent Spatial Memory Deficits in a Rat Model of Obstructive Sleep Apnea

**DOI:** 10.1371/journal.pone.0117990

**Published:** 2015-02-25

**Authors:** Chun-Sing Lam, George Lim Tipoe, Kwok-Fai So, Man-Lung Fung

**Affiliations:** 1 Department of Physiology, University of Hong Kong, Hong Kong, PR China; 2 Department of Anatomy, University of Hong Kong, Hong Kong, PR China; 3 Department of Ophthalmology, University of Hong Kong, Hong Kong, PR China; 4 Research Centre of Heart, Brain, Hormone & Healthy Aging, Li Ka Shing Faculty of Medicine, The University of Hong Kong, Hong Kong, PR China; 5 State Key Laboratory of Brain and Cognitive Science, Li Ka Shing Faculty of Medicine, The University of Hong Kong, Hong Kong, PR China; 6 Guangdong-HongKong-Macau Institute of CNS Regeneration, Jinan University, Guangdong, PR China; 7 Guangdong Key Laboratory of Brain Function and Diseases, Jinan University, Guangzhou 510632, China; Imperial College London, Chelsea & Westminster Hospital, UNITED KINGDOM

## Abstract

Chronic intermittent hypoxia (CIH) is a hallmark of obstructive sleep apnea (OSA), which induces hippocampal injuries mediated by oxidative stress. This study aims to examine the neuroprotective mechanism of *Lycium barbarum* polysaccharides (LBP) against CIH-induced spatial memory deficits. Adult Sprague–Dawley rats were exposed to hypoxic treatment resembling a severe OSA condition for a week. The animals were orally fed with LBP solution (1mg/kg) daily 2 hours prior to hypoxia or in air for the control. The effect of LBP on the spatial memory and levels of oxidative stress, inflammation, endoplasmic reticulum (ER) stress, apoptosis and neurogenesis in the hippocampus was examined. There was a significant deficit in the spatial memory and an elevated level of malondialdehyde with a decreased expression of antioxidant enzymes (SOD, GPx-1) in the hypoxic group when compared with the normoxic control. In addition, redox-sensitive nuclear factor kappa B (NFКB) canonical pathway was activated with a translocation of NFКB members (p65, p50) and increased expression levels of NFКB-dependent inflammatory cytokines and mediator (TNFα, IL-1β, COX-2); also, a significantly elevated level of ER stress (GRP78/Bip, PERK, CHOP) and autophagic flux in the hypoxic group, leading to neuronal apoptosis in hippocampal subfields (DG, CA1, CA3). Remarkably, LBP administration normalized the elevated level of oxidative stress, neuroinflammation, ER stress, autophagic flux and apoptosis induced by hypoxia. Moreover, LBP significantly mitigated both the caspase-dependent intrinsic (Bax, Bcl2, cytochrome C, cleaved caspase-3) and extrinsic (FADD, cleaved caspase-8, Bid) signaling apoptotic cascades. Furthermore, LBP administration prevented the spatial memory deficit and enhanced the hippocampal neurogenesis induced by hypoxia. Our results suggest that LBP is neuroprotective against CIH-induced hippocampal-dependent spatial memory deficits by promoting hippocampal neurogenesis and negatively modulating the apoptotic signaling cascades activated by oxidative stress and inflammation.

## Introduction

Obstructive sleep apnea (OSA) is a breathing disorder characterized by recurrent episodes of upper airway obstruction during sleep, resulting in severe oxygen desaturation interspersed with reoxgenation [1]. This is a highly prevalent disease affecting 5–7% populations in developed and developing countries [2]. Clinically, more than 70% OSA patients exhibit neurobehavioral impairment such as memory loss [3]. A substantial body of studies suggests that the neurocognitive deficits are closely associated with regional brain damages [4–6], particularly in the hippocampus with apoptosis possibly mediated by overproduction of reactive oxygen species (ROS) under chronic intermittent hypoxia (CIH) condition shown in experimental animals [7, 8]. Supporting this contention, ROS scavengers and pharmacological blockade of oxidative stress and inflammation could alleviate IH-induced apoptosis and spatial memory deficits in experimental animals [9–11]. Although markers of oxidative stress and inflammatory cytokines including tumor necrosis factor-alpha (TNF-α) and interleukins (ILs) are elevated in the serum of OSA patients and in the hippocampus of CIH animals [12, 13], the mechanistic role of oxidative stress and inflammation in the hippocampal injury is not fully depicted. It has been shown that ROS trigger neuronal cell death via an activation of caspase 3 [14], which could be regulated by pro-apoptotic protein Bax [15, 16]. Besides, caspase-dependent cascades are found to mediate neuronal apoptosis under various hypoxic paradigms, suggesting an involvement of caspase-dependent cascades induced by ROS and inflammation under hypoxic conditions [17–19].

Adult hippocampal neurogenesis is responsible for generating new neurons in the brain and found to be triggered by brain injuries and stress including hypoxia to replace damaged and malfunctioned neurons [20, 21]. Neurogenic BrdU-labeled and proliferative PCNA-labeled cells were increased in the mice hippocampus and primary hippocampal cultures following hypoxia or ischemia-induced hippocampal apoptosis, which may involve MAP kinase signaling pathway [22, 23]. Also, administration of hydrogen sulfide could restore hypoxia-induced cognitive impairment of mice by enhancing hypoxia-triggered hippocampal neurogenesis [24]. These observations indicate that the extent of neurogenesis initiated upon CIH-induced hippocampal injury may be crucial to restore the memory deficits of the rodents.

Apart from pharmacological intervention and conventional continuous positive airways pressure treatment for the OSA patients, alternative medicine with the use of herbal products has not been fully explored in its efficacy nor effectiveness against neurocognitive impairment. Wolfberry is the fruit of *Lycium barbarum* belonging to the family *Solanaceae* and is well known for the use as a healthy food supplement [25]. *Lycium barbarum* polysaccharides (LBP) are composed of structural complex of glycopeptides and are the most biologically active fraction of wolfberry [26]. Experimental studies have demonstrated anti-oxidative and anti-inflammatory properties of the beneficial effect of LBP in various disease models [27–29]. Administration of LBP has been shown to attenuate apoptosis of cortical neurons induced by glutamate or homocysteine [30, 31]; also it mitigated loss of retinal ganglion cells in a glaucoma model [32]. However, the neuroprotection of LBP against CIH-induced spatial memory deficits possibly caused by hippocampal apoptosis mediated by oxidative stress and inflammation is unclear. Recent studies showed that LBP could increase Bcl-2 expression, decrease the expression of apoptotic Apaf-1 and caspase-3 in the spleen of mice and restore Bax/Bcl-2 ratio spermatogenic cells of rat [33, 34], suggesting that the mechanistic effect of LBP may be exerted on caspase-mediated signaling cascades of apoptosis. In addition, LBP has been found to promote adult hippocampal neurogenesis inhibited by corticosterone and scopolamine in rodents [35, 36] and this implies that LBP may play a role in enhancing hippocampal regeneration under pathological conditions. The present study aimed to investigate the prophylactic effect of LBP administration against CIH-induced hippocampal-dependent spatial memory deficits in a rat model simulating a severe OSA condition in patients with an apnea-hypopnea index of 60; also the mechanistic effect of LBP on the intrinsic and extrinsic signaling cascades of apoptosis activated by ROS or inflammation, and hippocampal regeneration under CIH conditions.

## Materials and Methods

### 2.1 Materials and reagents

*Lycium barbarum* polysaccharides (LBP) was purchased from Hong Kong Institute of Biotechnology (Shatin, Hong Kong) and provided by Versitech Ltd. LBP powder was demonstrated to comprise about 35% arabinose, 16% galactose, 10% rhamnose together with small fractions of glucose, xylose, mannose, glucuronic acid and carotenoids by neutral sugar composition analysis. The purity of LBP was found to be ∼62% (w/w carbohydrates).

### 2.2 Ethics Statement

The use of animals in this study was conducted according to the requirements of the Cap. 340 Animals (Control of Experiments) Ordinance and Regulations, and all relevant legislation and Codes of Practice in Hong Kong. All the experimental and animal handling procedures were approved by the Faculty Committee on the Use of Live Animals in Teaching and Research in The University of Hong Kong (CULATR #2522–11).

### 2.3 Animals and hypoxic treatment

Adult Male Sprague Dawley rats (180–220g) were used and kept under standard condition in compliance with the requirements of The University of Hong Kong and the National Institute of Health with free access to animal chow and tap water. The Laboratory Animal Unit of The University of Hong Kong is fully accredited by the Association for Assessment and Accreditation for Laboratory Animal Care (AAALAC International). The animal room was controlled at a constant temperature (22±2°C), humidity and light: dark cycle (lights on 07:00–19:00 h). Animals were randomly divided into four experimental groups (6–8 rats in a group), namely normoxic non-treated control (Nx), LBP-treated normoxic groups (Nx+LBP), hypoxia-treated group (IH), LBP-treated hypoxic group (IH+LBP). The normoxic control rats were kept in room air whereas hypoxic rats were maintained in an acrylic chamber for normobaric hypoxia in the same room. Levels of oxygen in the chamber were altered from 21 to 5±0.5% per minute for 8 hours per day diurnally for 7 days. The desired oxygen levels were established by a mixture of room air and nitrogen and monitored by an oxygen analyzer (Vacumetrics Inc., St. Ventura, CA, USA). Before the daily hypoxic treatment, rats were orally fed with LBP solution in a dose of 1mg/kg with reference to a previous report [37]. The animals were anesthetized with halothane and then decapitated to harvest the hippocampus for experiments.

### 2.4 Malondialdehyde (MDA) level

Levels of MDA in hippocampal samples were determined by the use of a Bioxytech LPO-586 kit (OxisResearch, Portland, OR) according to the manufacture instruction. The absorbances of reaction products were read at 586 nm measured by spectrophotometry. Standard curves were constructed with 1,1,3,3-tetraethoxypropane as the standard. The MDA levels were normalized with corresponding protein amount determined by a Bio-Rad Protein Assay Kit (Bio-Rad, Hercules, CA) and the MDA value was expressed as μmol/mg.

### 2.5 Western Blot

The hippocampus was frozen in liquid nitrogen and then stored at −80°C. For the protein extraction, tissues were immersed in an ice-cold lysis buffer containing 1% Triton X-100, 1% Sodium deoxycholate, 0.1% SDS, 0.15 M sodium chloride, 0.01 M sodium phosphate and protease inhibitor cocktail (Sigma, P2714) at the final pH of 7.4 followed by mechanical homogenization. The homogenates were then incubated at 4°C for 2 h with shaking to enhance protein solubilization. The insoluble materials were removed by centrifugation at 13,000 ×*g* for 15 min at 4°C, supernatant enriched with proteins were collected and protein concentration was determined using protein assay (Bio-Rad Laboratories, USA). Equal amount of protein from each sample was resolved by electrophoresis on a 10% or 15% polyacrylamide gel as appropriate. The proteins on the gel were electrically transferred on a PVDF membrane (Bio-Rad). After incubating with 5% non-fat skim milk for 1 h, the membrane was probed with primary antibody in TBST (100mM Tris–HCl, pH 7.5, 0.9% NaCl, 0.1% Tween 20) overnight at 4°C with gentle agitation: the specific primary antibodies against SOD-1, SOD-2, GPx-1 and IL-1β (rabbit polyclonal; 1:1000, 1:1000, 1:500 and 1:250 respectively; Santa Cruz Biotechnology, CA, USA), against Bax and Bcl-2 (rabbit polyclonal; 1:500 and 1:1000 respectively; Santa Cruz Biotechnology, CA, USA), against cleaved caspase-3 (Asp175), cleaved caspase-8 (Asp387), cytochrome-c, p-JNK (Thr183/Tyr185) and total JNK (rabbit polyclonal; 1:1000; Cell Signaling Technology), against GRP78/Bip, p-PERK (Thr980), total PERK, p-I kappa B α (Ser32), total I kappa B, p-Akt (Ser473), total Akt, p-cJun (Ser63), PTEN, CHOP, Lamin B1, Beclin-1, Atg 12, Atg 3, p62, LC3B (rabbit polyclonal; 1:500; Cell Signaling Technology), against TNF-α, COX-2 (goat polyclonal; 1:100 and 1:200 respectively; Santa Cruz Biotechnology, CA, USA), against NFκB p65 and NFκB p50 (mouse polyclonal; 1:250 and 1:250 respectively; Santa Cruz Biotechnology, CA, USA, against PCNA and cyclin D1 (mouse monoclonal; 1:500; Cell Signaling Technology and against FADD, BDNF, Bid, LAMP-1 (rabbit polyclonal; 1:500, 1:200,1:3000 and 1:500 respectively, Millipore, CA, USA) were used in Western Blotting. The membrane was washed with TBST and incubated with anti-rabbit secondary antibody (for SOD-1, SOD-2, GPx-1 IL-1β, Bax, Bcl-2, cytochrome C, cleaved caspase-3, FADD, Bid, cleaved caspase-8, p-JNK, total JNK, p-I kappa B α, total I kappa B α, p-Akt, total Akt, p-cJun, BDNF, PTEN, GRP-78/Bip, p-PERK, total PERK, CHOP, Beclin-1, Atg 12, Atg 3, p62, LAMP-1 and LC3B) and anti-goat secondary antibody (for TNF-α, COX-2) and anti-mouse secondary antibody (for PCNA, cylcin D1, NFκB p65 and NFκB p50) (1:10000 dilution in TBST) for 2 h at room temperature. Beta-actin (Monoclonal; 1:10000; Santa Cruz Biotechnology, CA, USA) was used as an internal control for whole cell lysate and cytosolic fraction while Lamin B1 (Rabbit polyclonal) was employed as an internal control for nuclear fraction. After washing off the unbound antibody with TBST, the expression of the antibody linked protein was determined by an ECLTM Western Blotting Detection Reagents (Millipore). The optical density of the bands was measured and quantified by Image J (National Institute of Health, MD, USA). The optical density of protein products was expressed as arbitrary units.

### 2.6 Histology

For histological experiments, rats were firstly anesthetized with halothane and decapitated to harvest hippocampi. Tissues harvested were either fixed by 10% Neural Buffered Formalin (NBF) or 4% paraformaldehyde (PFA). Tissues fixed in NBF required 3 days for thorough fixation and then was dehydrate by the increasing magnitude of reagent-graded ethanol (70%, 80%, 90%, 95% and 100%). After dehydration, tissue would be immersed in chloroform overnight followed by paraffin embedment. Serial sections of 4 μm thickness were cut and mounted on silanized slides (DAKO, Denmark). Sections were kept in the oven overnight at 37°C.

On the other hand, transcardial perfusion was performed with the use of saline followed by 4% PFA for whole body fixation. Tissues harvested were post-fixed in 4% PFA for 1 day followed by 30% sucrose solution immersion until the tissues sank to the bottom. Tissue would then be embedded in OCT for frozen section and store at-80°C for further experiments.

### 2.7 Apoptosis

The terminal deoxynucleotidyl transferase-biotin dUTP-nick end labeling (TUNEL) assay was used to detect 3’ hydroxyl ends in fragmented DNA in the hippocampus. In brief, after dewaxation and rehydration, tissue sections were processed according to the manufacturer’s instructions for the TUNEL assay with the *in situ* cell death detection kit (Roche, USA) and then stained with fast red dye. Sections treated with DNase I recombinant were used as the positive control. Labeling solution without terminal transferase was used in place of TUNEL reaction mixture for negative control. The positive TUNEL staining cells in dentate gyrus, subfields CA1 and CA3 were counted under a high-power magnification (20X) field of light microscope (Zeiss Axiolab, Carl Zeiss Inc. Germany). At least five fields were sampled in a section and data were expressed as the number of TUNEL-positive counts.

### 2.8 Immunohistochemistry

Immunohistochemical staining of proliferating cellular nuclear antigen (PCNA) was performed to reveal proliferating cells in hippocampus. In brief, 3% hydrogen peroxide was added on deparaffinized formalin fixed sections of hippocampus to block endogenous peroxidase activity followed by immersing in antigen retrieval solution (0.1 M citric acid buffer, pH 6.0) for 10 min at 98°C and trypsinized buffer for 6 min at room temperature. To block non-specific binding of anti-serum, sections were incubated in 20% normal horse serum for 2 hours. Sections were then incubated with primary antibodies of PCNA (mouse polyclonal antibody; 1:2000 dilutions; Cell signaling Technology) in 0.05M Tris-HCl buffer containing 2% bovine serum albumin overnight at 4°C. Sections were washed three times in PBS, and then incubated with biotinylated link agent and streptavidin peroxidase of LSAB kit (K0690, DAKO) for 30 min at room temperature. After the rinsing, peroxidase was visualized by adding in 0.05% diaminobenidine (DAB) containing 0.03% hydrogen peroxide in Tris-HCl buffer (pH7.5) for 3–5 min. Sections were washed in distilled water and counterstained with hematoxylin. Positive staining was indicated by a brown color. Control sections stained with normal mouse IgG as are uniformly negative. The positive PCNA staining cells in dentate gyrus, subfields CA1 and CA3 were revealed under a high-power magnification (100X) of light microscope (Zeiss Axiolab, Carl Zeiss Inc. Germany). The counting method of PCNA-positive count was same as TUNEL assay.

### 2.9 BrdU injection paradigm and immunohistochemistry

BrdU (Roche) was dissolved in PBS. Rats were received BrdU solution intraperitoneal injection (50mg/kg) at days 5, 6 and 7. Rats were sacrificed 2 hours after the last injection. Frozen sections were used for BrdU (Roche) immunohistochemistry study to label and quantify the proliferating cells in hippocampus. Sections were immersed in the phosphate buffered saline (PBS) for 5 min and in antigen retrieval solutions followed by blocking in normal horse serum. BrdU antibody was diluted in the incubation buffer provided by the manufacturer kit (1:10) and incubated with the sections at 37°C. The binding of antibody was visualized with the help of LSAB kit and DAB staining. The positive BrdU-labeled cells in dentate gyrus, subfields CA1 and CA3 were counted under a high-power magnification (10X and 40X) of light microscope (Zeiss Axiolab, Carl Zeiss Inc. Germany). The counting method of BrdU-positive count was same as TUNEL assay. In order to identify the phenotypes of those proliferating cells, double immunofluorescent staining was performed. NeuN, Iba-1 and GFAP were used as markers of mature neurons, activated microglial cells and astrocytes respectively. Similar to immunohistochemical staining, sections was immersed in antigen retrieval sections and blocked in normal horse serum. Then, sections were incubated in the serum containing anti-BrdU mouse monoclonal antibody diluting with incubation buffer (1:10) provided by the manufacturer kit (Roche) at 37°C for 30 min. Then, the sections were rinsed with PBS for 5 min twice. Secondary anti-mouse antibodies Alexa-fluor 488 (Life technologies) was added to incubate the section at 37°C for 30 min. After that, the sections were rinsed with PBS for 5 min twice. Other primary anti-rabbit polyclonal antibodies NeuN (1:400; Millipore), GFAP or Iba-1(1:500 and 1:300 respectively; Novus Biologicals, U.S.A.) were added to incubate the section at 4°C overnight. Again, sections were rinsed with PBS for 5 min twice. Secondary anti-rabbit antibodies Alexa-fluor 594 (Life technologies) was added to incubate the section at room temperature for 60 min. Sections incubated with only secondary antibodies served as the negative control. The sections were then rinsed with PBS for 5 min twice. Confocal laser scanning microscopy (Carl Zeiss LSM 510 Meta/Axiocam) was used to image the fluorescent signals.

### 2.10 Water Morris Maze Behavioral Test

Spatial learning memory was assessed by Morris water maze behavioral test which was taken for two weeks in our study. Behavioral testing comprised a standard place-training reference memory task in the water maze in order to train the rats to locate the submerged hidden platform with the only use of distal spatial cues. Rats were placed in a pool (180 cm in diameter, filled to a depth of 50 cm, and maintained at 25 ± 1°C) and had to find a platform hidden below the water surface (made opaque with non-toxic food dye) using visual cues in the room. The pool was divided into four quadrants (1, 2, 3, 4) and the hidden platform was placed in quadrant 4. In the training session, from day 1 to day 6, a maximum of 90s was given to the rats to search the platform where it was allowed 30s for resting. At day 7, first probe test trial with the hidden platform removed that last for 90s was given to assess the training-based spatial memory acquired by the rats. Hypoxic treatment with or without LBP administration was given to the rats at day 8 to day 14. Second probe test trial was given again to examine the spatial memory of the rats. Track path, percentage of time spent and total distance swam by the rats were recorded and analyzed by video camera, interfaced with the video tracking system (TopScan Version 3.00, Clever, U.S.A.) and path tracing software (CorelDRAW X4, Corel Cooperation).

### 2.11 Statistical analysis

Data from each group were expressed as mean ± SEM. Statistical comparisons among groups were performed using One way ANOVA followed by Tukey’s post-hoc test for multiple comparisons. A *p*<0.05 was considered to be statistically significant with the use of Graphpad Prism software (Graphpad Software, Inc., San Diego, USA)

## Results

### 3.1 LBP reversed CIH-induced spatial memory deficits

**Fig 1:**
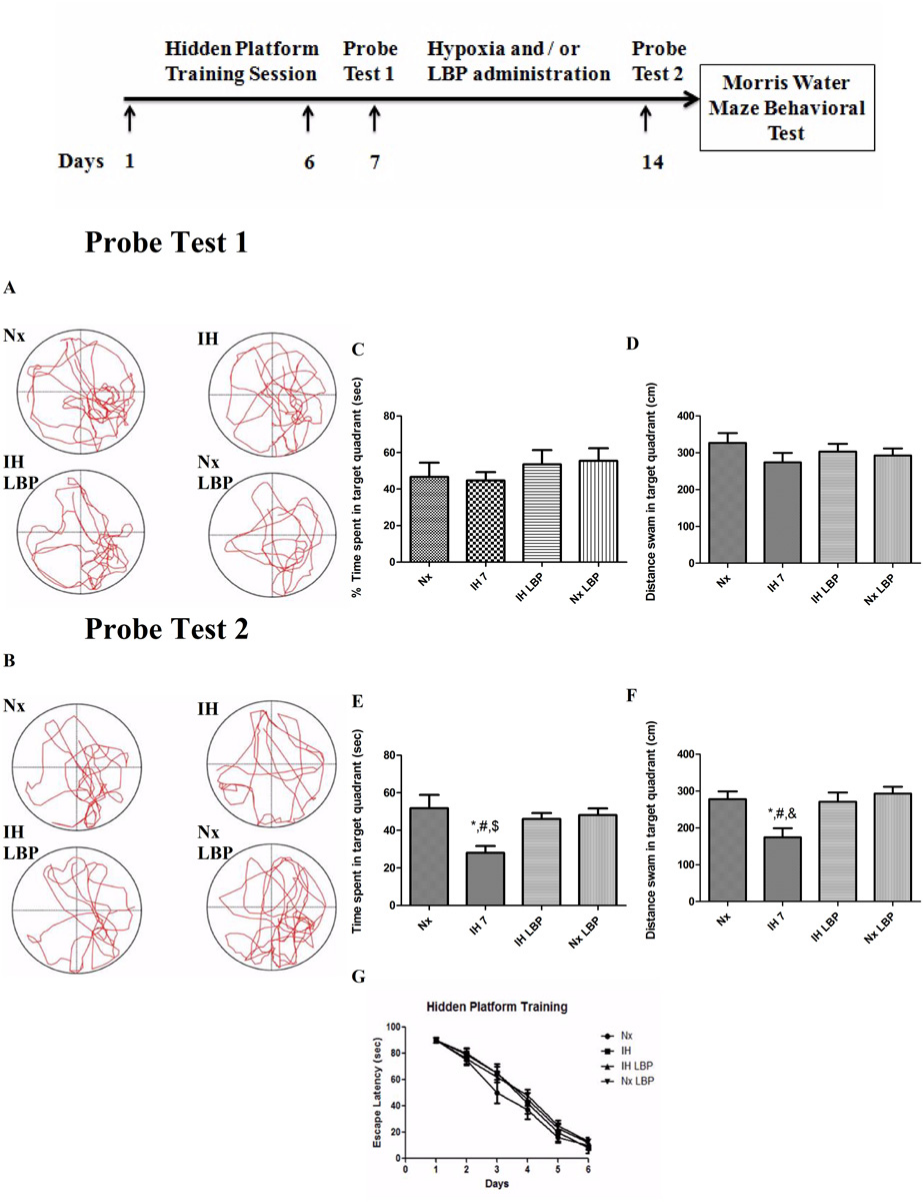
CIH treatment caused remarkable spatial memory loss in rats, which was significantly reversed by LBP administration. Panels A and B represent the tracking paths of the rats in normoxic (Nx) or hypoxic (IH) groups; LBP-treated hypoxic (IH+LBP) or normoxic (Nx+LBP) groups in probe tests 1 and 2, respectively. Panel C, D, E and F summarize the percentage of time spent and distance swam in target quadrant of trained rats. The escape latency of the rats from all groups decreased along the training session (panel G). Data are mean ± SEM (n = 12). *p < 0.05 when compared with Nx, ^#^p < 0.05 when compared with IH+LBP, ^$^p < 0.05 when compared with Nx+LBP groups.

The escape latency of the rats from all groups (n = 12) decreased along the training session. In probe test 1, there was no significant difference observed neither in the percentage of time spent nor in the total distance swam by the rats among all the groups in the target quadrant (Fig. 1). In probe test 2, there was a significant decrease in the percentage of time spent and also the total distance swam in the target quadrant in the hypoxic-treated group (28 ± 3.6%, 174.3 ± 25cm) when comparing to the control (51.8 ± 7.1%, 278.2 ± 21cm). These values in the hypoxic group treated with LBP (46 ± 3.2%, 271.3 ± 25cm) were not different from the normoxic group (Fig. 1). Results strongly supported that LBP could prevent CIH-induced spatial memory deficits.

### 3.2 LBP prevented hippocampal apoptosis induced by hypoxia

**Fig 2:**
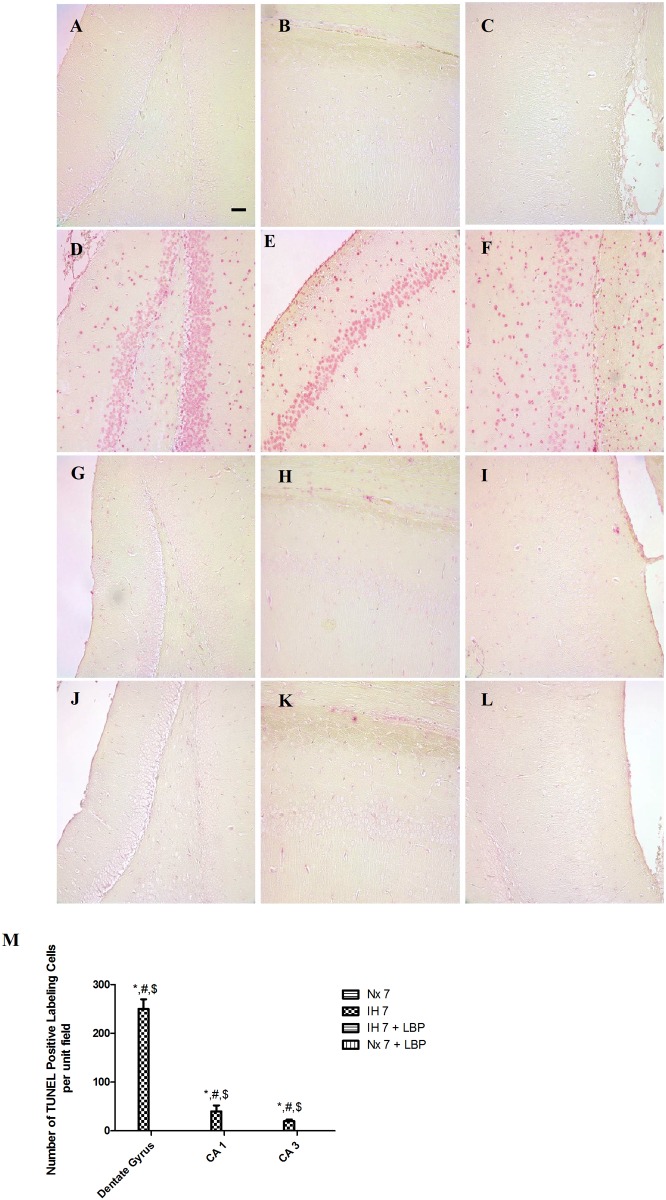
Administration of LBP prevented CIH-induced apoptosis in the dentate gyrus (DG, panels A, D, G, J), CA1 (panels B, E, H, K) and CA3 (panels C, F, I, L) subfields of the hippocampus, respectively, for the normoxic (Nx7) or hypoxic (IH7) groups; LBP-treated hypoxic (IH7+LBP) or normoxic (Nx7+LBP) groups. Panel M summarizes the number of TUNEL-positive counts in hippocampal subfields of the normoxic and hypoxic groups with or without LBP pre-treatment. Data presented are expressed as mean ± SEM (n = 6–8). *p < 0.05 comparing to Nx7, ^#^p < 0.05 comparing to IH7+LBP, ^$^p < 0.05 comparing to Nx7+LBP groups. Magnification: 20×; Scale Bars: 50μm.

In the hypoxic group, the number of TUNEL-positive cells was remarkably more in the dentate gyrus, CA1 and CA3 subfields of the hippocampus than those of the control (n = 6–8). There were more apoptotic cells in dentate gyrus than the CA1 and CA3 subfields. In contrast, there were no TUNEL-positive cells found in the LBP-treated groups and the normoxic control (Fig. 2). Results strongly suggested that pretreatment of LBP could prevent CIH-induced hippocampal apoptosis.

### 3.3 LBP mitigated intrinsic and extrinsic cascades of apoptosis

**Fig 3:**
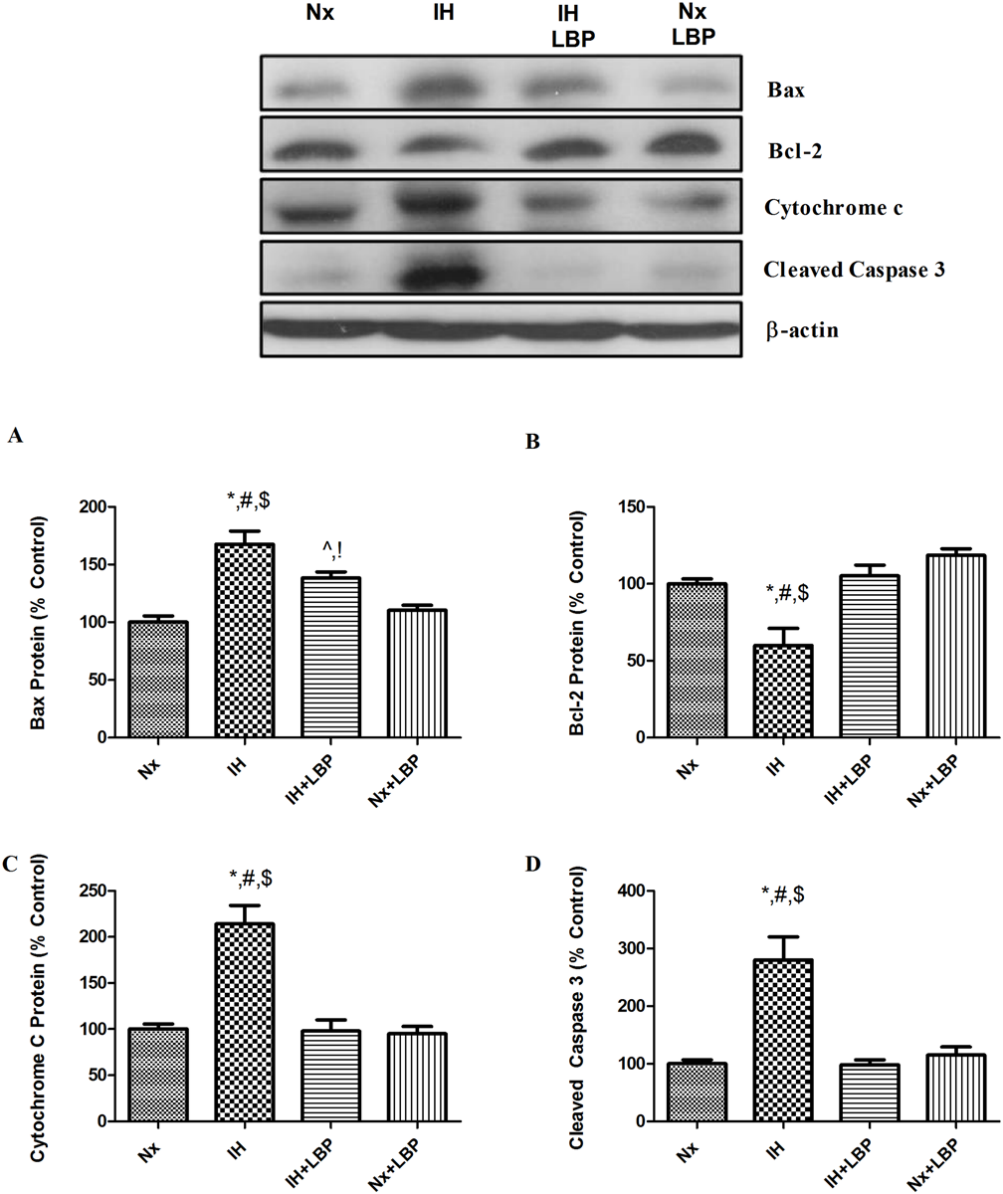
LBP mitigated intrinsic caspase-dependent apoptosis induced by CIH treatment. Levels of the protein expression (upper panel) of (A) Bax, (B) Bcl-2, (C) cytochrome-c and (D) cleaved caspase-3 in the hippocampus of the normoxic (Nx) or hypoxic (IH) groups; LBP-treated hypoxic (IH+LBP) or normoxic (Nx+LBP) groups are summarized. β-actin was an internal control. Data are mean ± SEM (n = 6–8). For Bax, *p < 0.05 when compared with Nx, ^#^p < 0.05 when compared with IH+LBP, ^$^p < 0.05 when compared with Nx+LBP groups, ^^^p < 0.05 when compared with Nx, ^!^p < 0.05 when compared with Nx+LBP. For Bcl-2, cytochrome-c and cleaved caspase 3, *p < 0.05 when compared with Nx, ^#^p < 0.05 when compared with IH+LBP, ^$^p < 0.05 when compared with Nx+LBP groups.

**Fig 4:**
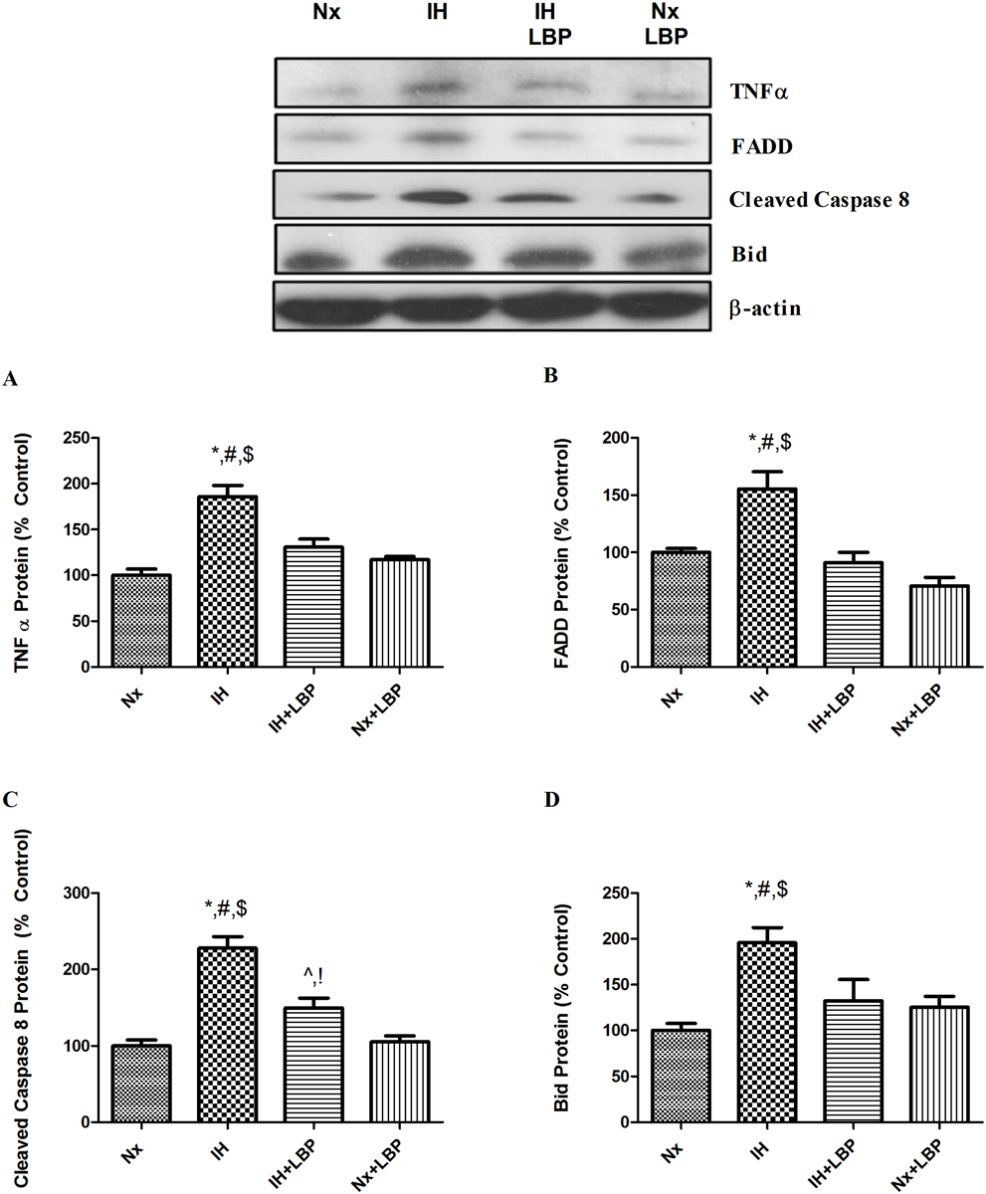
LBP ameliorated extrinsic caspase-dependent apoptosis induced by CIH treatment. Levels of the protein expression (upper panel) of (A) TNFα, (B) FADD, (C) cleaved caspase 8 and (D) Bid in the hippocampus of the normoxic (Nx) or hypoxic (IH) groups; LBP-treated hypoxic (IH+LBP) or normoxic (Nx+LBP) groups are summarized. β-actin was an internal control. Data are mean ± SEM (n = 6–8). For TNFα, FADD and Bid, *p < 0.05 when compared with Nx, ^#^p < 0.05 when compared with IH+LBP, ^$^p < 0.05 when compared with Nx+LBP groups. For cleaved caspase 8, *p < 0.05 when compared with Nx, ^#^p < 0.05 when compared with IH+LBP, ^$^p < 0.05 when compared with Nx+LBP groups, ^^^p < 0.05 when compared with Nx, ^!^p < 0.05 when compared with Nx+LBP.

To investigate the mechanistic effect of LBP on CIH-induced hippocampal apoptosis, we examined both the caspase-dependent intrinsic and extrinsic signaling cascades of apoptosis. For the intrinsic pathway, the expression of pro-apoptotic protein Bax was significantly increased by 70% in the hypoxic group when compared with that of the control (n = 6–8), which was significantly lowered in the LBP-treated hypoxic group. In contrast, the expression of anti-apoptotic protein Bcl-2 was significantly decreased by 40% in the hypoxic group but was restored by the LBP pre-treatment. In addition, levels of intrinsic apoptotic proteins cytochrome-c and cleaved caspase-3 were markedly elevated by 2–3 folds in the hypoxic group, but were normalized in the LBP-treated group (Fig. 3). Furthermore, levels of apoptotic proteins of the extrinsic signaling cascade, namely, TNF-α, FADD and Bid were significantly increased, respectively, by about 80%, 60% and 200% in the hypoxic group, which were greatly suppressed by the LBP administration. Also, the level of cleaved caspase-8 was doubled in the hypoxic group when comparing to the control (n = 6–8), which was significantly lowered in the LBP-treated group (Fig. 4).

### 3.4 LBP attenuated CIH-induced oxidative stress

**Fig 5:**
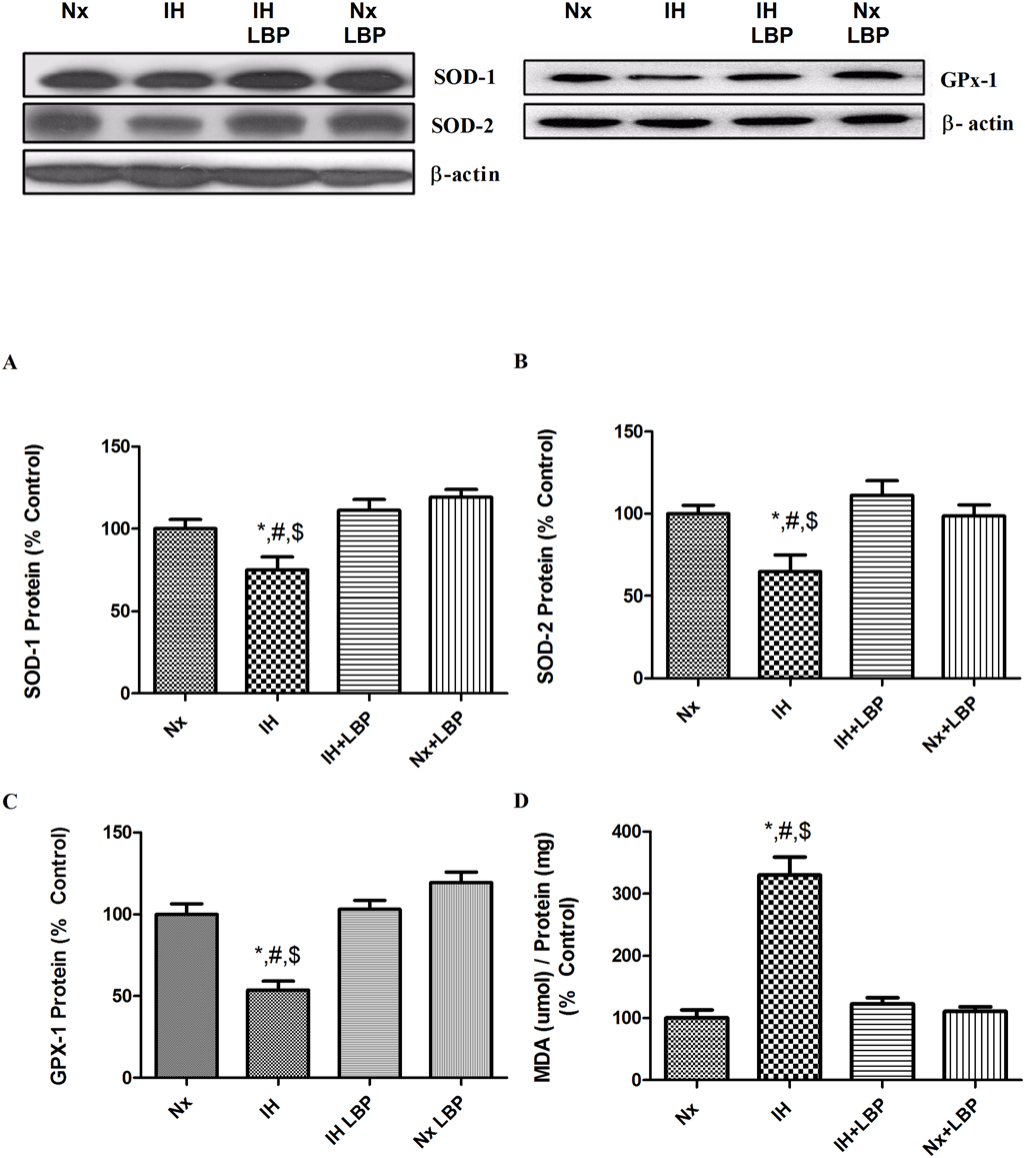
LBP attenuated CIH-induced oxidative stress. Levels of the protein expression (upper panel) of (A) SOD-1, (B) SOD-2, (C) GPx-1 and (D) MDA content in the hippocampus of the normoxic (Nx) or hypoxic (IH) groups; LBP-treated hypoxic (IH+LBP) or normoxic (Nx+LBP) groups are summarized. β-actin was an internal control. Data are mean ± SEM (n = 6–8). *p < 0.05 when compared with Nx, ^#^p < 0.05 when compared with IH+LBP, ^$^p < 0.05 when compared with Nx+LBP groups.

The MDA level in the hippocampus of the hypoxic group was remarkably increased by 3 folds when compared with that of the control group (n = 6–8). The elevated MDA level was normalized in the LBP-treated group. In addition, the expression of Cu/Zn-SOD (SOD-1), Mn-SOD (SOD-2) and GPx-1 was significantly lowered, respectively, by 30%, 40% and 50% in the hypoxic group but was neutralized by the LBP administration (Fig. 5).

### 3.5 LBP antagonized CIH-induced inflammation through suppression of redox-sensitive nuclear factor kappa B (NFКB) canonical pathway in the hippocampus

**Fig 6:**
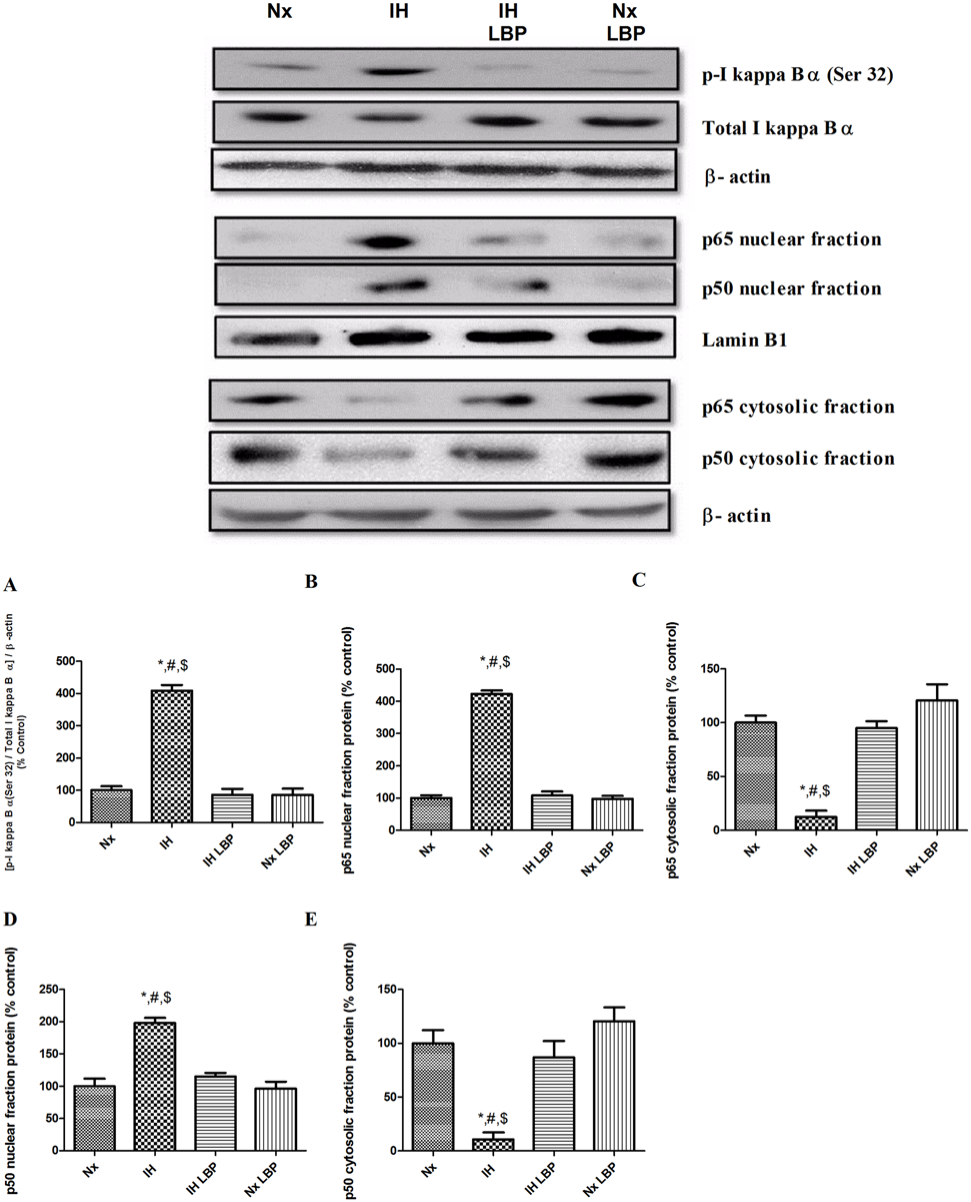
LBP ameliorated CIH-induced inflammation by inhibiting the degradation of redox-sensitive NFКB canonical pathway negative regulator IКB and by suppressing the translocation of NFКB member p65 and p50 from cytosol to nucleus. Levels of (A) phosphorylation of IКB α, nuclear protein expression of (B) p65, (C) cytosolic protein expression of (C) p65, nuclear protein expression of (D) p50, and cytosolic protein expression of (E) p50 in the hippocampus (upper panel) of the normoxic (Nx) or hypoxic (IH) groups; LBP-treated hypoxic (IH+LBP) or normoxic (Nx+LBP) groups are summarized. Lamin B1 was an internal control of the nuclear fraction, whereas β-actin was an internal control of the cytosolic fraction and whole cell lysate. Data are mean ± SEM (n = 6–8). *p < 0.05 when compared with Nx, ^#^p < 0.05 when compared with IH+LBP, ^$^p < 0.05 when compared with Nx+LBP groups.

**Fig 7:**
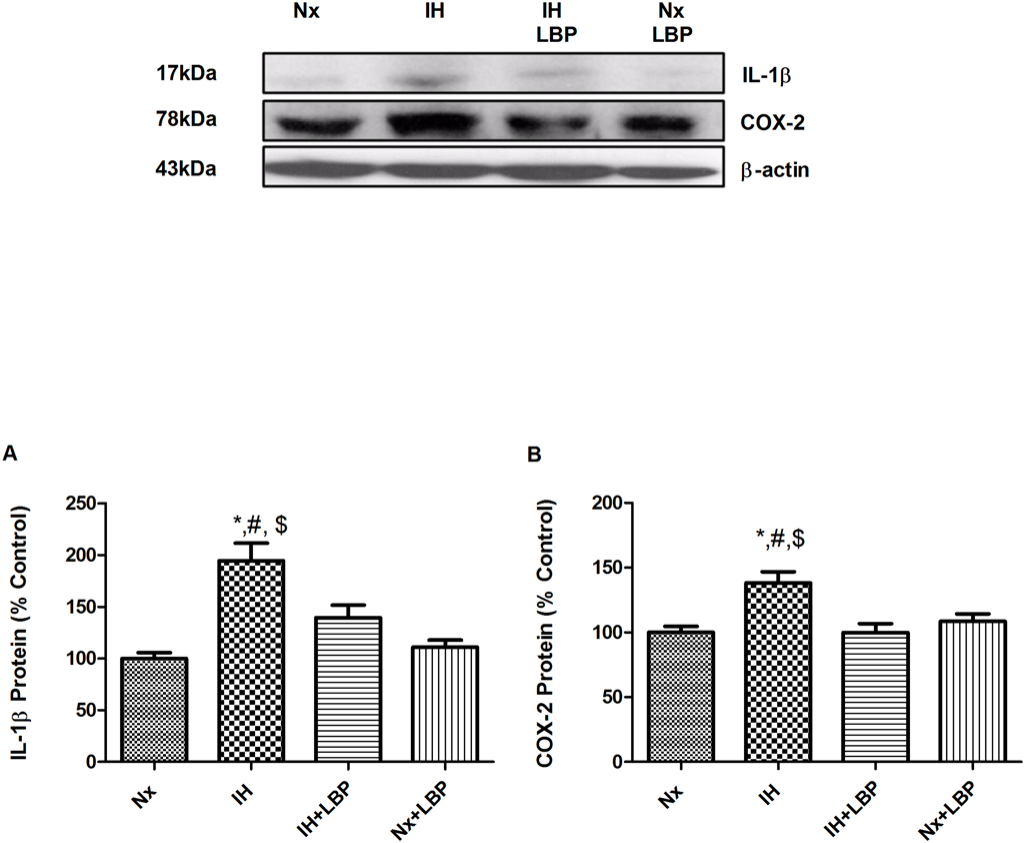
LBP downregulated NFКB-dependent inflammatory cytokine and mediator. Levels of the protein expression (upper panel) of (A) IL-1β and (B) COX-2 in the hippocampus of the normoxic (Nx) or hypoxic (IH) groups; LBP-treated hypoxic (IH+LBP) or normoxic (Nx+LBP) groups are summarized. β-actin was an internal control. Data are mean ± SEM (n = 6–8). *p < 0.05 when compared with Nx, ^#^p < 0.05 when compared with IH+LBP, ^$^p < 0.05 when compared with Nx+LBP groups.

Inhibitor of Kappa B alpha (I*К*Bα), the negative regulator of NF*К*B canonical pathway, was highly phosphorylated with a markedly decreased amount of total I*К*B in the hypoxic treated group (n = 6–8). Also, the ratio of p- IκB α / total IκB α was about 4 folds increased when compared to that of the control. Besides, nuclear fraction expressions of NFκB p65 and p50 were significantly increased by 4 folds and 2 folds respectively, and the cytosolic fractions expressions of NFκB p65 and p50 were significantly decreased by 80% and 90% respectively. These changes were normalized by LBP pre-treatment illustrated in Fig. 6. In addition, there was a significant increase in the expression of inflammatory cytokine IL-1β and mediator cyclooxyenase-2 (COX-2), respectively, by 94% and 40%, in the hippocampus of the hypoxic group. The increased expression of IL-1β and COX-2 was largely antagonized in the LBP-treated group (Fig. 7).

### 3.6 LBP reduced CIH-induced endoplasmic reticulum (ER) stress in the hippocampus but not through autophagic flux promotion

**Fig 8:**
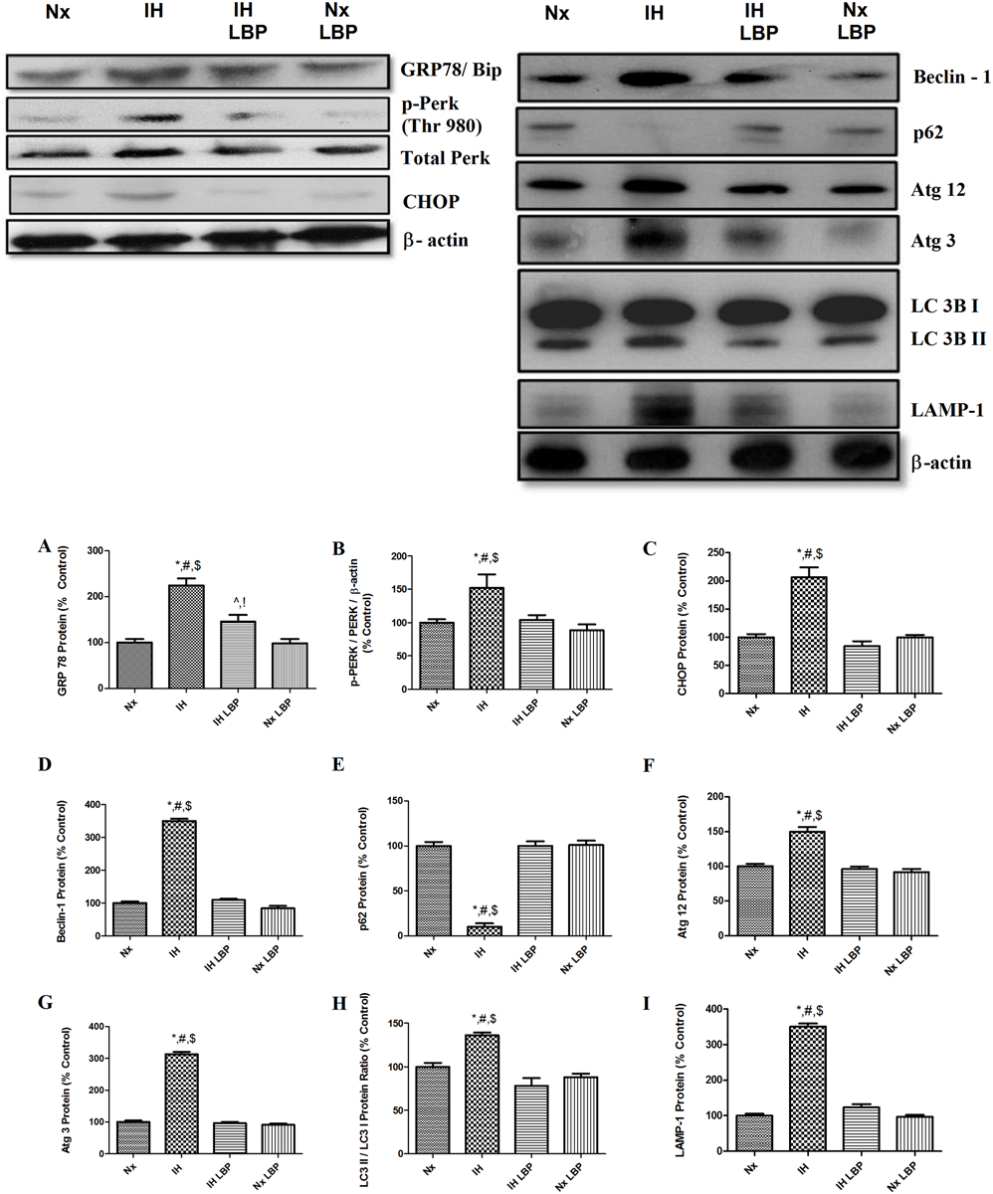
LBP alleviated CIH-induced ER stress in the hippocampus but not through autophagic flux. Levels of the protein expression (upper panel) of (A) GRP78, (B) phosphorylation of PERK, (C) CHOP, (D) Beclin-1, (E) p62, (F) Atg12, (G) Atg3, (H) LC3 II/I ratio and (I) LAMP-1 in the hippocampus of the normoxic (Nx) or hypoxic (IH) groups; LBP-treated hypoxic (IH+LBP) or normoxic (Nx+LBP) groups are summarized. β-actin was an internal control. Data are mean ± SEM (n = 6–8). For GRP78, *p < 0.05 when compared with Nx, ^#^p < 0.05 when compared with IH+LBP, ^$^p < 0.05 when compared with Nx+LBP groups, ^^^p < 0.05 when compared with Nx, ^!^p < 0.05 when compared with Nx+LBP. For p-PERK, CHOP, Beclin-1, p62, Atg12, Atg 3, LC3II/I and LAMP-1, *p < 0.05 when compared with Nx, ^#^p < 0.05 when compared with IH+LBP, ^$^p < 0.05 when compared with Nx+LBP groups.

ER stress sensors chaperone GRP78/Bip, PERK and downstream effector CHOP were significantly increased by about 2 folds, 1.5 folds and 2 folds respectively in the hypoxic group when compared with those of controls (n = 6–8) but were greatly alleviated by LBP administration (Fig. 8).

Protein levels of autophagic markers Beclin-1, Atg 12, Atg 3, LC3 II/LC3I ratio and LAMP-1 were significantly elevated by 3.5, 0.5, 3, 0.3 and 3.5 folds in the hypoxic group when compared with those of controls (n = 6–8). In contrast, p62 was degraded (90%) in the hypoxic treated group when comparing to the control group. Importantly, LBP administration normalized all the autophagic markers to the basal level (Fig. 8).

### 3.7 LBP facilitated CIH-triggered hippocampal neurogenesis but not astrocytic and microglial regeneration

**Fig 9:**
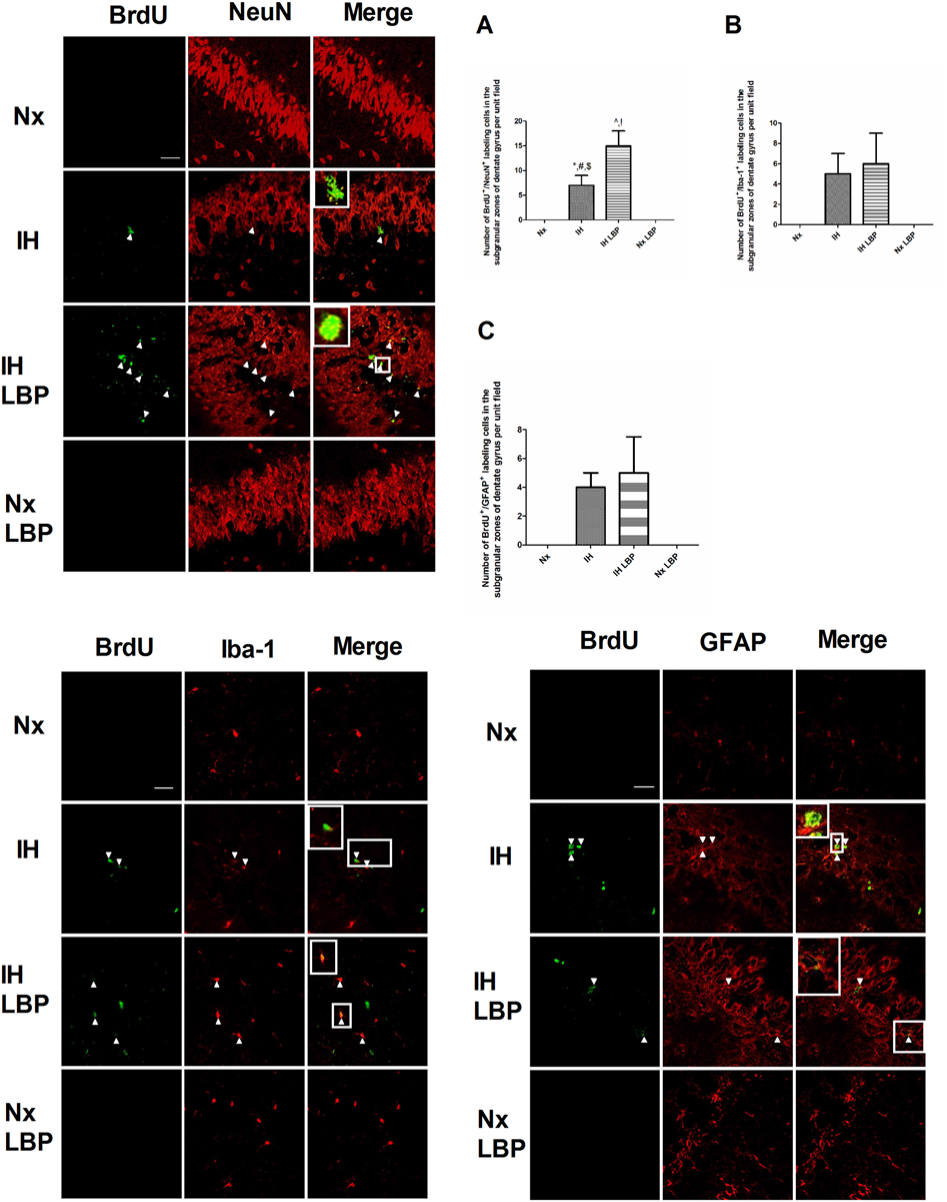
LBP facilitated CIH-induced neurogenesis but not the regeneration of astrocytic and microglia in the SGZ of dentate gyrus of the hippocampus. Number of BrdU^+^NeuN^+^ counts in the hippocampal SGZ was further increased by the LBP administration in hypoxia-treated rats. Panels A, B and C summarize the number of BrdU^+^NeuN^+^, BrdU^+^Iba-1^+^ and BrdU^+^GFAP^+^ respectively. Magnification: 63×; Scale bar: 100μm.

The number of BrdU ^+^/NeuN^+^ positive labeled cells was markedly more in hypoxic treated groups than those of the control (n = 6–8). LBP administration further augmented the number of BrdU ^+^/NeuN^+^ positive labeled cells in hypoxic treated groups. On the contrary, there was no BrdU ^+^/NeuN^+^ positive labeled cells found in LBP-treated groups and the normoxic control (Fig. 9). The numbers of BrdU ^+^/GFAP^+^ and BrdU ^+^/Iba-1^+^ were significantly increased in hypoxic treated groups comparing with those of the control. However, LBP administration did not further increase the number of BrdU ^+^/GFAP^+^ and BrdU ^+^/Iba-1^+^ positive labeled cells in hypoxic treated groups. In contrast, there was no BrdU ^+^/GFAP^+^ and BrdU ^+^/Iba-1^+^ positive labeled cells found in LBP-treated groups and the normoxic control (Fig. 9).

### 3.8 LBP enhanced CIH-activated hippocampal regeneration through Akt/PCNA axis

**Fig 10:**
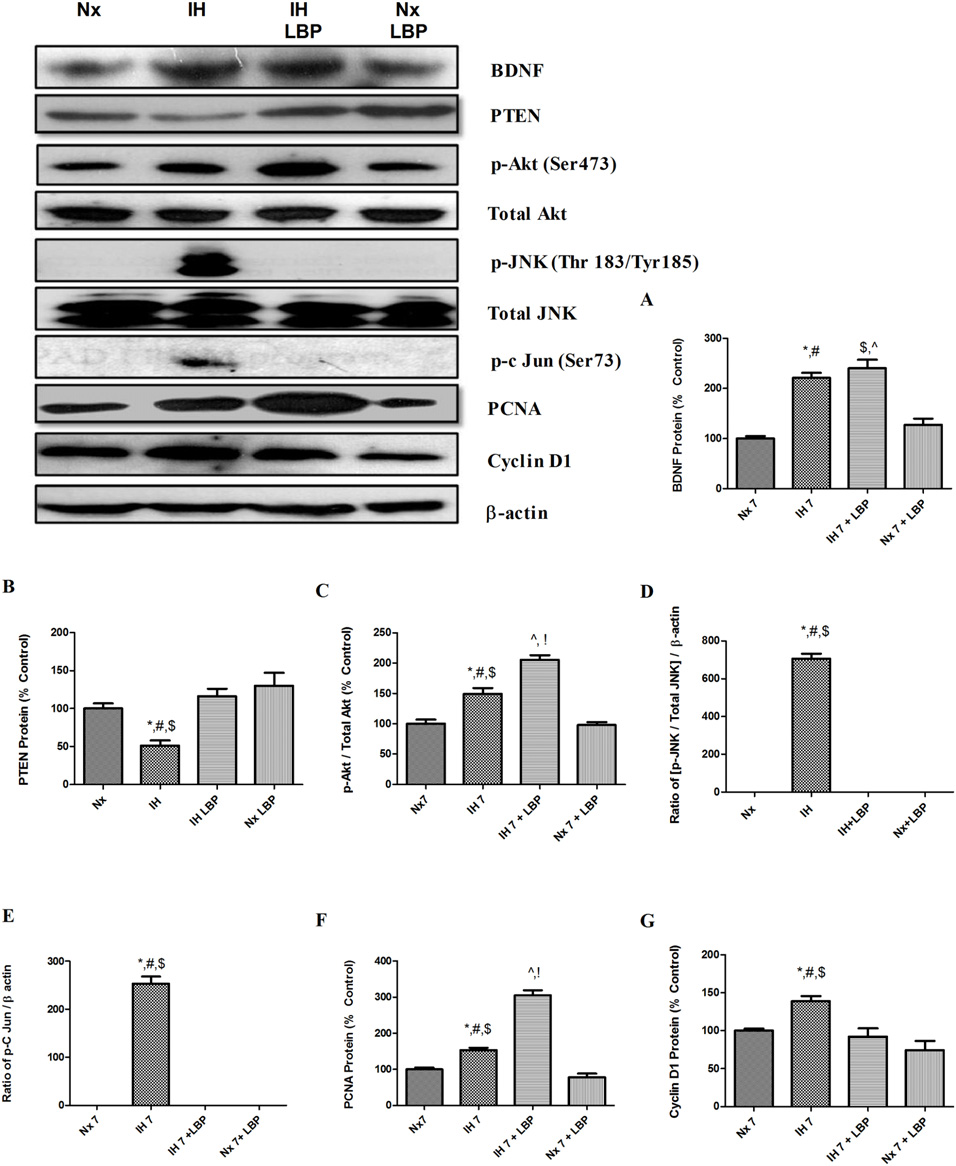
LBP facilitated CIH-activated hippocampal regeneration through Akt/PCNA axis. Levels of the protein expression (upper panel) of (A) BDNF (B) PTEN, (C) p-Akt, (D) p-JNK, (E) p-c Jun, (F) PCNA and (G) cyclin D1 in the hippocampus of the normoxic (Nx) or hypoxic (IH) groups; LBP-treated hypoxic (IH+LBP) or normoxic (Nx+LBP) groups are summarized. For BDNF, *p < 0.05 when compared with Nx groups, ^#^p < 0.05 when compared with Nx+LBP groups, ^$^p < 0.05 when compared with Nx groups, ^^^p < 0.05 when compared with Nx +LBP groups. For PTEN, p-JNK, p-c Jun and cyclin D1, *p < 0.05 when compared with Nx, ^#^p < 0.05 when compared with IH+LBP, ^$^p < 0.05 when compared with Nx+LBP groups. For Akt and PCNA, *p < 0.05 when compared with Nx groups, ^#^p < 0.05 when compared with IH+LBP groups P, ^$^p < 0.05 when compared with Nx+LBP groups, ^^^p < 0.05 when compared with Nx, ^!^p < 0.05 when compared with Nx+LBP groups.

To unravel the mechanistic effect of LBP on CIH-activated hippocampal regeneration, we investigated the proteins level of proliferation markers (PCNA and cyclin D1), phosphorylation of Akt (Ser 473) and JNK/SAPK (Thr183/Tyr185). For proliferative markers, the expressions of PCNA and cyclin D1 were found significantly elevated by 50% and 40% respectively in the hypoxic group when compared with that of the control (n = 6–8). The level of PCNA expression was further increased by LBP administration by about 2 folds. In addition, levels of phosphorylation of Akt and JNK were markedly increased by 50% and 7 folds in the hypoxic group when compared with that of the control. Administration of LBP elevated further the phosphorylated Akt by 50% but normalized the phosphorylation of JNK to the control level (Fig. 10).

**Fig 11:**
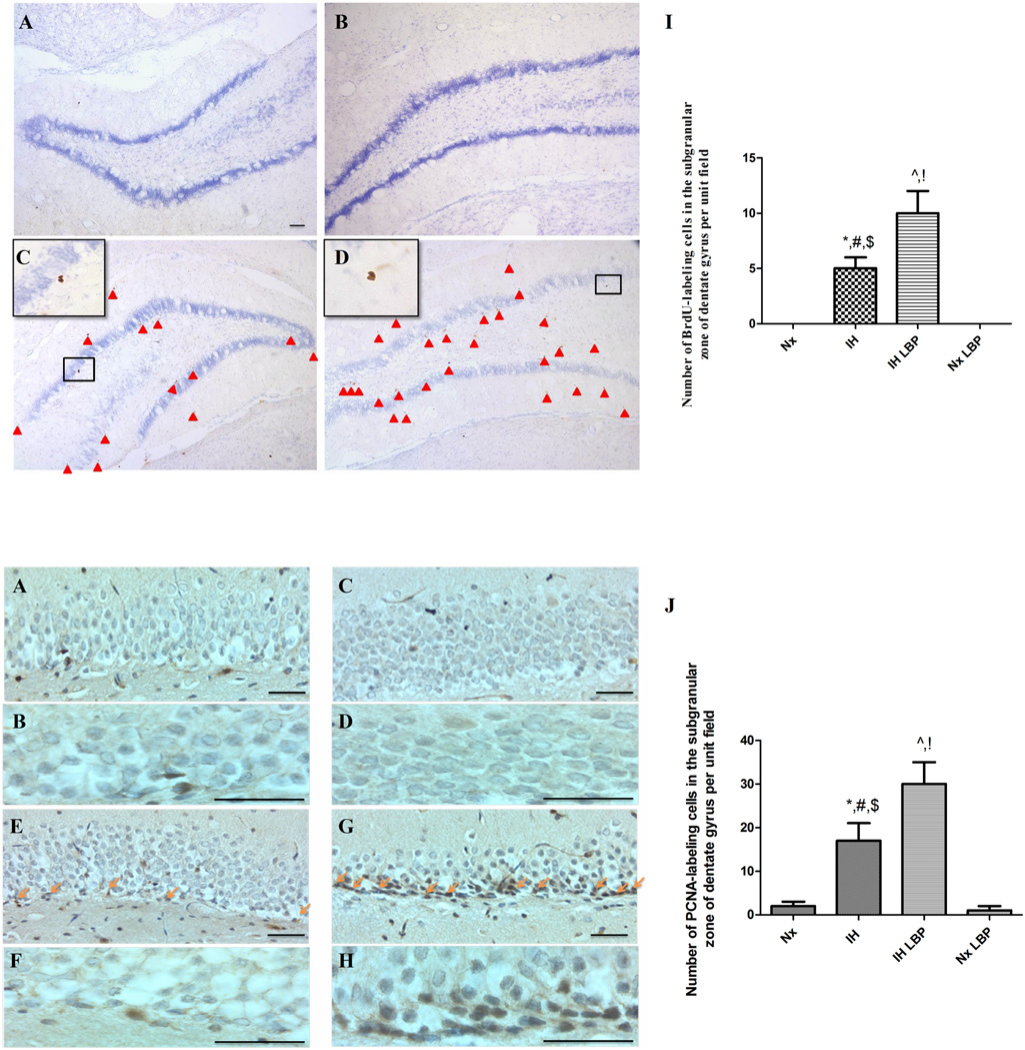
LBP enhanced CIH-induced proliferative activities in the hippocampus. Panels A and B summarize the number of positive BrdU- or PCNA-staining cells in the SGZ of dentate gyrus in the hippocampus among different groups. Data are mean ± SEM (n = 6–8). *p < 0.05 when compared with Nx, ^#^p < 0.05 when compared with IH+LBP, ^$^p < 0.05 when compared with Nx+LBP groups, ^^^p < 0.05 when compared with Nx, ^!^p < 0.05 when compared with Nx+LBP. Magnification: 10× and 40× (BrdU), Scale bar: 100μm; 40× and 100× (PCNA), Scale Bars: 50μm.

Protein level of upstream mediators of Akt pathway including BDNF and PTEN were also examined. BDNF was significantly up-regulated by 2 folds in the hypoxic-treated and LBP co-treated hypoxic groups when compared with that of the control (n = 6–8). However, there was no significant difference observed between these two groups. The protein level of PTEN was remarkably degraded (50%) in the hypoxic-treated group when compared with that of controls but was significantly restored by the LBP administration (Fig. 10). Moreover, immunohistochemical staining of neurogenic marker BrdU and proliferative marker PCNA were performed. Consistently, numbers of BrdU and PCNA positive labeled cells were more in the hypoxic group than in that of the control. Administration of LBP further increased the number of BrdU and PCNA labeled cells in the SGZ of dentate gyrus in the hippocampus (Fig. 11).

## Discussion

This is the first report showing the neuroprotective mechanism of LBP against hippocampal-dependent spatial memory deficits resulting from apoptosis mediated by oxidative stress and inflammation under intermittently hypoxic conditions resembling the neurobehavioral impact of a severe apnea-hypopnea in OSA patients. Importantly, our results demonstrated the mechanistic involvement of both intrinsic and extrinsic signaling cascades of apoptosis, upon which the impact was completely neutralized by the LBP treatment; also, CIH-induced hippocampal neurogenesis was enhanced by LBP administration through the Akt/PCNA signaling cascade. Thus, the neuroprotective effect of LBP against CIH-induced memory deficits with minimal perturbation in healthy rats supports the efficacy and prophylactic use of herbal product LBP in the prevention of neurocognitive impairment in OSA patients. In this context, the rodent model used in this study simulated the hypoxic condition with an apnea-hypopnea index of 60 in the patient. Consequently, the hypoxia-treated rat developed significant neurobehavioral impairment accompanied with remarkable elevated levels of oxidative stress, inflammation and apoptosis in the hippocampus, highlighting the major clinical manifestations in the brain of OSA patients. This is also in consistent with growing bodies of evidence suggesting that OSA-induced cognitive deficit is due to a significant loss of neurons in the hippocampus, which is the critical structure for spatial memory and learning and is highly sensitive to intermittently hypoxic challenges [38, 39]. The effective dose LBP is only 1mg/kg in animal study [40, 41] which is equivalent to 0.25g of dried wolfberry for a 60-kg individual [27]. It is reasonable and convenient for daily consumption to retard the onset of neurocognitive impairment of OSA patients.

We found that anti-oxidative and anti-inflammatory properties of LBP are crucial to the neuroprotection against CIH-induced spatial memory deficits. Lipid membrane of organelles chemically reacts with ROS and generates free radicals leading to lipid peroxidation and protein nitration, which forms the end product MDA. We found that the MDA level was significantly elevated in the hippocampus of hypoxic rats, which was completely normalized by the LBP pre-treatment. This is in consistent with the result of previous reports, suggesting an important role of ROS overproduction in CIH-induced oxidative stress, which could also be ameliorated by ROS scavengers or antioxidants [8, 9]. In addition, the level of endogenous antioxidant enzymes, notably SOD-1, SOD-2 and GPx-1, was significantly decreased in hypoxia and was also markedly restored by LBP. Results are in line with a previous report showing that LBP could enhance the antioxidant capacities against oxidative stress *in vivo* and *in vitro* [42–45]. These results indicate that LBP could modulate the level of ROS and antioxidant enzymes to antagonize CIH-induced oxidative stress.

In addition to oxidative stress induced by CIH, free radicals induce tissue injuries leading to inflammation which is prominent in OSA patients and CIH animals. This is confirmed by the elevated expressions of proinflammatory cytokines (TNFα, IL-1β) and mediator (COX-2) in the hippocampus of hypoxic rats, which is consistent with the finding of previous reports [8, 46]. Also, we found that the expression of inflammatory cytokines is dependent on NFКB activation. In effect, stimulation of cytokine receptors including TNFαR1 signals the extrinsic cascade of apoptosis, which is mediated by FADD protein signaling the activation of caspase-8 with self-cleavage [47]. Subsequently, activated caspase-8 cleaves its substrate Bid that forms a complex with Bax in the mitochondrial membrane for inhibiting the anti-apoptotic effect of Bcl-2 causing the release of cytochrome-c [48]. Our results show significant elevated levels of FADD, cleaved caspase-8 and Bid in the hippocampus of the hypoxic rat, strongly suggesting an activation of the extrinsic signaling cascade. In fact we found an increased activity of JNK with phosphorylation at residues Thr183 and Tyr185 in the hypoxic group, which is known to be required for TNF-α induced apoptosis [49, 50]. Thus, this is an important mechanistic pathway underlying the impact of CIH-induced inflammation on hippocampal apoptosis. Alternatively, increased ROS level could also increase the activity of Bid and sufficiently induce apoptosis which has been shown in primary hippocampal neurons [51, 52].

Importantly, the CIH-induced inflammation was greatly suppressed by the LBP pre-treatment. In this regard, LBP has been shown to attenuate hepatic inflammation induced by carbon tetrachloride [41]. The anti-inflammatory effect of LBP could explain the deactivation of the extrinsic signaling cascade of apoptosis because of the decreased production of inflammatory cytokines. Also inflammatory mediators activated by the injury and ROS could aggravate the vicious cycle among oxidative stress, inflammation and tissue injury [53]. Thus the suppressive effect of LBP on the inflammatory cascade is an important component of the neuroprotective mechanism against CIH-induced hippocampal injury.

Endoplasmic reticulum is a subcellular organelle to fold protein properly and highly susceptible to hypoxia challenges [54]. In addition, autophagy, a conserved cytoprotective mechanism, is activated to maintain the cellular homeostasis. We found increased expressions of ER stress sensor proteins (GRP78/BIP, PERK, CHOP) with an increased autophagic flux in the hippocampus of the hypoxic rats. Interestingly LBP administration could completely normalize the expression of ER sensor proteins but did not affect the autophagic flux. These results suggest that autophagy was responsive to the ER stress induced by CIH but might not be involved in the neuroprotective effect of LBP against spatial memory deficits. This is probably due to the restoration of Bcl-2, an inhibitor that hinders Beclin-1 to participate in the initiation of autophagic processes [55]. Also, the absence of ER stress observed in LBP-treated hypoxic group is in line with the finding with normalized autophagic flux in the LBP-treated groups.

Apoptosis is tightly associated with oxidative stress [56]. It has been proposed that ROS are crucial mediators of neuronal apoptosis induced by intermittent hypoxia [4]. In neurons, apoptosis activated by oxidative stress is regulated by pro-apoptotic protein Bax and anti-apoptotic protein Bcl-2. It has been shown that increases in the ratio between Bax and Bcl-2 signal the intrinsic cascade of apoptosis [57, 58]. Upon stress stimuli, Bax is shown to destabilize and rupture the mitochondrial membrane, leading to the release of cytochrome-c from the inner membrane and the formation of apoptosome which subsequently activates and cleaves caspase-3 to initiate apoptosis. This cascade is negatively regulated by Bcl-2 because it inhibits the release of cytochrome-c [59]. We found that the ratio between Bax and Bcl-2 was significantly elevated in the hippocampus of the hypoxic rat, suggesting an activation of the intrinsic signaling cascade. In fact the levels of cytochrome-c and cleaved caspase-3 were more than doubled in the hypoxic group with a remarkable level of apoptosis. These findings strongly support that oxidative stress mediates the CIH-induced hippocampal apoptosis via the activation of intrinsic cascade.

**Fig 12:**
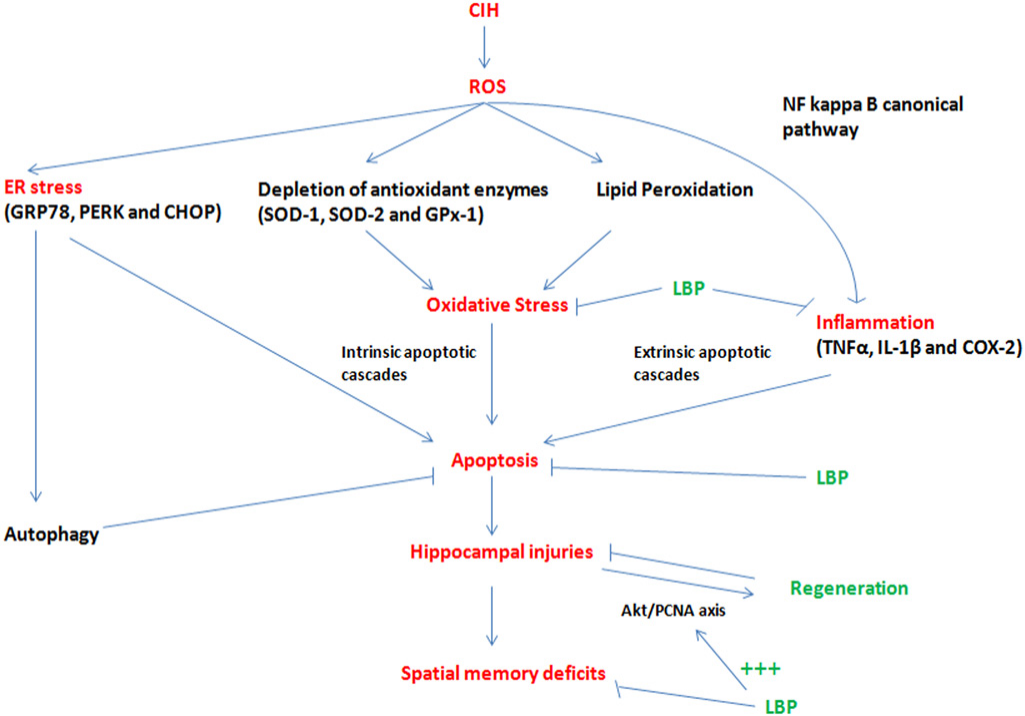
Summary of neuroprotection mechanisms of LBP against spatial memory deficits induced by CIH.

We and other have shown that the CIH-induced apoptosis could be ameliorated by antioxidants, despite the unknown mechanism [8, 9]. Under CIH condition, ROS are massively produced at a greater rate than ROS being removed in the CNS. As a result, oxidative stress occurs, and in turn depleting the antioxidant enzymes and ultimately triggering the oxidative damages to lipids, nucleic acid and protein. These oxidative damages are observable in our study. Upon these damages, the downstream proinflammatory cytokines such as TNF α and IL-1β are produced. Meanwhile, redox-sensitive canonical NFКB are also activated. The activation of NFКB and the release of proinflammatory cytokines, which are NFКB-dependent, will synergistically cause neuronal damages and deaths indicated by augmented cleaved caspase 3 expression) through extrinsic apoptotic cascades. On the other hand, ROS causes mitochondrial dysfunction and also trigger neuronal death through intrinsic apoptotic cascade. In the present study, we found that LBP could increase the levels of endogenous antioxidant enzymes (SOD and GPx-1), and help to balance the ratio of ROS and antioxidant enzymes in the cells. Therefore, LBP can protect the cells against oxidative stress induced by CIH and also inhibit oxidative stress-induced inflammation. Most importantly, LBP could significantly mitigate both the caspase-dependent intrinsic and extrinsic signaling cascades of apoptosis, providing evidence for the neuroprotective mechanism of LBP by which alleviates the impacts of ROS and inflammation induced by CIH. Thus, the levels of Bax, Bcl-2, cleaved caspase-3, cytochrome-c were normalized in the LBP-treated group. Our findings also give support to the observation that LBP could maintain the level of Bax-to-Bcl-2 ratio for survival in cardiomyocytes upon ischemic/reperfusion insults [60]. In addition, we found that the extrinsic cascade (FADD, cleaved caspase-8, Bid) and elevated JNK activity were significantly abrogated by LBP administration. This is in consistent with the observation that LBP ameliorates the level of phosphorylated JNK and apoptosis induced by homocysteine in cultured cortical neurons [30]. Thus, it is likely that JNK mediates the effect of TNF-α on CIH-induced apoptosis via the extrinsic cascade. Furthermore, the neuroprotective mechanism is also explained by the fact that LBP pre-treatment substantially neutralized CIH-induced oxidative stress and inflammation in the hippocampus (Fig. 12).

Upon stress-induced damages, intrinsic regenerative mechanisms activate adult hippocampal neurogenesis as part of the recovery process. In fact, we found both BrdU and PCNA-positive labeled cells and the protein levels of PCNA and cyclin D1 were significantly increased in the hippocampus of the hypoxic rat. Intriguingly, LBP administration could increase further the number of positive-labeled cells and protein levels of PCNA in the hypoxic group. This is also shown by double immunofluorescent labeling of the regenerative cells with different cellular markers NeuN, GFAP and Iba-1 respectively for mature neurons, astrocytes and activated microglial cells. We found that there was a significant increased number of NeuN^+^/BrdU^+^ cells but not the GFAP^+^/BrdU^+^ or Iba-1^+^/BrdU^+^ cells among hypoxia-treated and LBP co-treated hypoxic groups. These results are in consistent with our contention that LBP promoted CIH-induced hippocampal neurogenesis, which could replace malfunctioned neurons and facilitate the recovery of neurobehavioral deficit.

Concerning the mechanism that underpins the LBP effect on promoting CIH-induced hippocampal neurogenesis, the signaling cascade mediated by Akt, which is an activator of PCNA, has been shown to protect hippocampal neurons against apoptosis [61]. The activity of Akt is represented by the extent of phosphorylation at Ser 473, which was significantly increased in the hypoxic group and was further enhanced by the LBP administration. These results are in consistent with the report showing that LBP could increase Akt activity in primary cortical neurons upon β-amyloid toxicity. Indeed, the expression of upstream mediators of Akt survival pathway BDNF was markedly elevated with a significant decreased PTEN level in the hippocampus of the hypoxic rat. Administration of LBP selectively restored the degraded PTEN level but not affecting the BDNF expression. In this regard, given that LBP could ameliorate the increased JNK activity in the hippocampus of the hypoxic rat, JNK could play dual roles in mediating TNFα-induced apoptosis and also in activating regeneration through the JNK/c-Jun/cyclin D1 pathway under CIH conditions (Fig. 11). Nevertheless, the upstream mediators of Akt pathway targeted by LBP still await further investigation. Alternatively, IGF-1, another potent Akt activator, is a possible candidate as it is recently found transiently increased by LBP in cultured cortical neurons [62]. The mechanistic effects of LBP against spatial memory deficits induced by CIH are summarized in the Fig. 12.

Our CIH model used in this study mimics the episodic oxygen desaturation in OSA patients during sleeping, which is a significant cause of the pathophysiological consequence of OSA by inducing adverse effects including oxidative stress, neuroinflammation and neuronal loss in the rats’ brains. However, other clinical manifestations in OSA patients, such as non-rapid eye movement sleep (NREM) predominance and sleep fragmentation may not be simulated in this CIH model. Nevertheless, this study mainly focuses on the prophylactic effects of LBP against OSA-induced neurocognitive deficits. In future, therapeutic effects of LBP will be assessed with the use of another protocol with longer exposure and LBP treatment application after CIH-induced pathological features.

## Conclusion

We have demonstrated the neuroprotective mechanism of LBP against CIH-induced spatial memory deficits by antagonizing oxidative stress, inflammation and hippocampal apoptosis via mitigation of the intrinsic and extrinsic signaling cascades, and by enhancing hippocampal neurogenesis. Importantly, these works suggest that LBP may be proposed as a herbal supplement for preventing neurological deficits in OSA patients.
